# *Lagenaria siceraria* fruit: A review of its phytochemistry, pharmacology, and promising traditional uses

**DOI:** 10.3389/fnut.2022.927361

**Published:** 2022-09-16

**Authors:** Muhammad Saeed, Muhammad Sajjad Khan, Kinza Amir, Jannat Bi Bi, Muhammad Asif, Asadullah Madni, Asghar Ali Kamboh, Zahid Manzoor, Umair Younas, Sun Chao

**Affiliations:** ^1^College of Animal Science and Technology, Northwest A&F University, Yangling, China; ^2^The Cholistan University of Veterinary and Animal Sciences, Bahawalpur, Pakistan; ^3^Mayo Hospital Lahore, Lahore, Pakistan; ^4^Department of Physical Education, Beijing Sports University, Beijing, China; ^5^District Head Quarter (DHQ) Hospital, Vehari, Pakistan; ^6^Department of Pharmaceutics, Faculty of Pharmacy, The Islamia University of Bahawalpur, Bahawalpur, Pakistan; ^7^Department of Veterinary Microbiology, Sindh Agriculture University, Tando Jam, Pakistan

**Keywords:** *Lagenaria siceraria* fruit, phytochemistry, pharmacology, nutritional potential, pharmacological effect

## Abstract

Since ancient times, the Cucurbitaceae family is used as a therapeutic option in human medicine. This family has around 130 genera and 800 species. Researchers have studied the various plants of this family including *Lagenaria siceraria* due to their medicinal potential. Various properties are beneficial for human health, that have been attributed to *L. siceraria* like antioxidant, hypolipidemic, diuretic, laxative, hepatoprotective, analgesic, antihypertensive, cardioprotective, central nervous system stimulant, anthelmintic, free radical scavenging, immunosuppressive, and adaptogenic. The fruit of this plant is commonly used as a vegetable that has a low-calorie value. The species possess a diverse set of biological compounds like flavonoids, sterols, saponins, and terpenoids. Vitamins, choline, flavonoids, minerals, proteins, terpenoids, and other phytochemicals are also found in the edible parts of this plant. Besides 17 different amino acids, many minerals are reported to be present in the seeds of *L. siceraria*. According to the USDA nutritional database per 100 g of *L. siceraria* contains 14 Kcal energy, 3.39 g carbohydrates, 0.62 g protein, 0.2 g fat, and 0.5 g fiber. *L. siceraria* performs a wide range of pharmacological and physiological actions. The literature reviewed from various sources including PubMed, Science Direct, Google scholar, etc. shows the remarkable potential to treat various human and animal illnesses due to its' potent bioactive chemicals. The key objective of this thorough analysis is to present a summary of the data about the beneficial and harmful effects of *L. siceraria* intake on human health, as well as in veterinary fields.

## Introduction

There is an emergent concern about herbal medicines across the world, which is complemented by more laboratory research into the pharmacological characteristics of bioactive substances and their capacity to cure various disorders. Through ethnopharmacology and traditional medicine, a slew of new medications has made their way onto the worldwide market (1). For centuries, herbal treatments have been used to cure and manage a variety of ailments. Herbal medications are a viable alternative to current synthetic treatments owing to their few adverse effects and are regarded as safe and useful in the treatment of human ailments. The Cucurbitaceae family includes the *Lagenaria siceraria* (Mol.) Standley fruit (Bottle gourd), utilized in a separate system of traditional medicine to cure numerous ailments (2). A significant number of medicinally beneficial plants belong to the Cucurbitaceae family. This family has around 130 genera and 800 species. Cucurbitacin, a secondary metabolite found in the seeds and fruit sections of several cucurbits, has been described to have purgative, emetic, and antihelmintic actions. This category of chemicals had been considered for its anti-inflammatory, hepatoprotective, cytotoxic, and cardiovascular properties (3). Bottle gourd (*L. siceraria*), family Cucurbitaceae is a medicinal plant whose diverse sections had been identified for their therapeutic potential. The plant's fruiting body is well-liked for its taste and extraordinary nutritional content, which includes practically all of the needed ingredients for good health. The plant might provide physiologically active polysaccharides (4). Being a domestic plant, it is for both food and medicine. Cardioprotective, antidepressant, anti-hyperglycemic, antimicrobial, cytotoxic, anti-inflammatory, antihyperlipidemic, anti-urolithiasis, antianxiety, analgesic, anticancer, diuretic, anthelmintic, antihepatotoxic, anthelmintic, antistress, immunomodulatory, antiulcer, hepatoprotective, and antioxidant activities have been studied in various parts of this plant. To emphasize the medicinal value of this plant, its phytochemical elements, and traditional, pharmacological, and medicinal applications are studied in this review. This would be beneficial in resurrecting its relevance and highlighting its many potential qualities to motivate academics to do more study on *L. siceraria* (2). The key objective of this thorough analysis is to present a summary of the data about the beneficial and harmful effects of *L. siceraria* intake on human health, as well as in veterinary fields like the poultry and livestock sector.

## Botanical description

The *L. siceraria* (Molina) is a member of the Cucurbitaceae family and is also called Bottle gourd. It is a climbing perennial plant that is extensively grown as a vegetable crop in tropical nations such as Thailand, Egypt, India, Japan, and the rest of the world (1, 5). The fruits of Bottle gourd have a variety of shapes: they can be huge and rounded, small and bottle-shaped, or slim and serpentine, and they can grow to be over a meter long. Rounder varieties are typically called calabash gourds.

**Figure 1:**
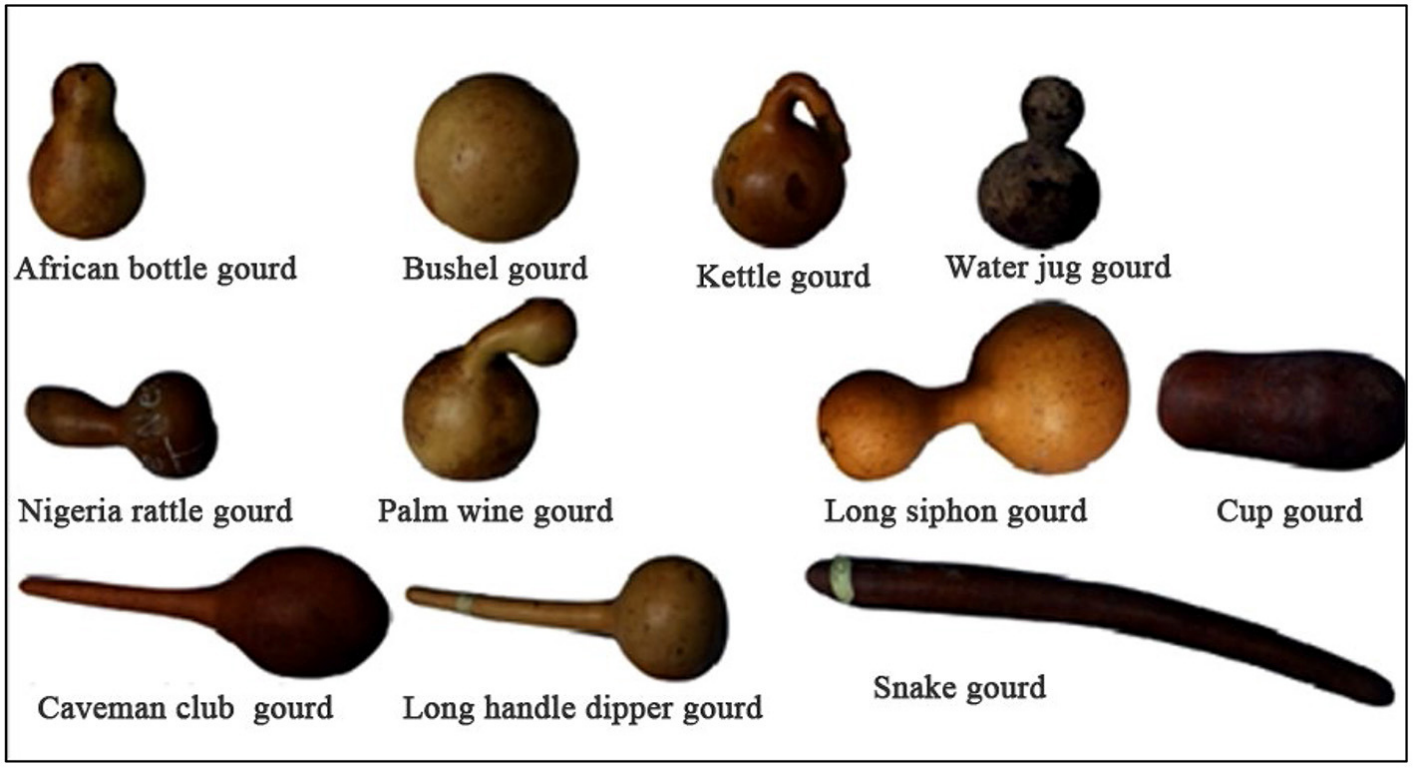
Different shapes of *Lagenaria siceraria* found across the world (6).

The fruit diversity and phytogeographical distribution of *L. siceraria* in Nigeria were studied and 24 different shapes of fruits were explored as shown in Figure 1 (6).

The bottle gourd is said to have originated in Africa and spread worldwide in pre-Columbian times, maybe *via* floating on the oceans. It moved from India to Indonesia, New Zealand, and China where it has diversified into several local kinds. It is a robust annual vine with huge leaves and a lush look that may be grown as a running or climbing vine (7) (Table 1).

**Table 1:** Botanical description of various parts of *Lagenaria siceraria* (7).

**Botanical description**	
Vine	Branched and climbs along the stem
Leaves	15 inches wide, circular, smooth margins, broad lobes, velvety texture
Foliage	Covered with soft hairs and on crushing gives a foul musky odor
Flowers	Borne singly on the axils of the leaves, white and attractive, 4 inches in diameter, spreading petals
Seeds	Brownish, rectangular in shape, have grooved notches near the end

**Figure 2:**
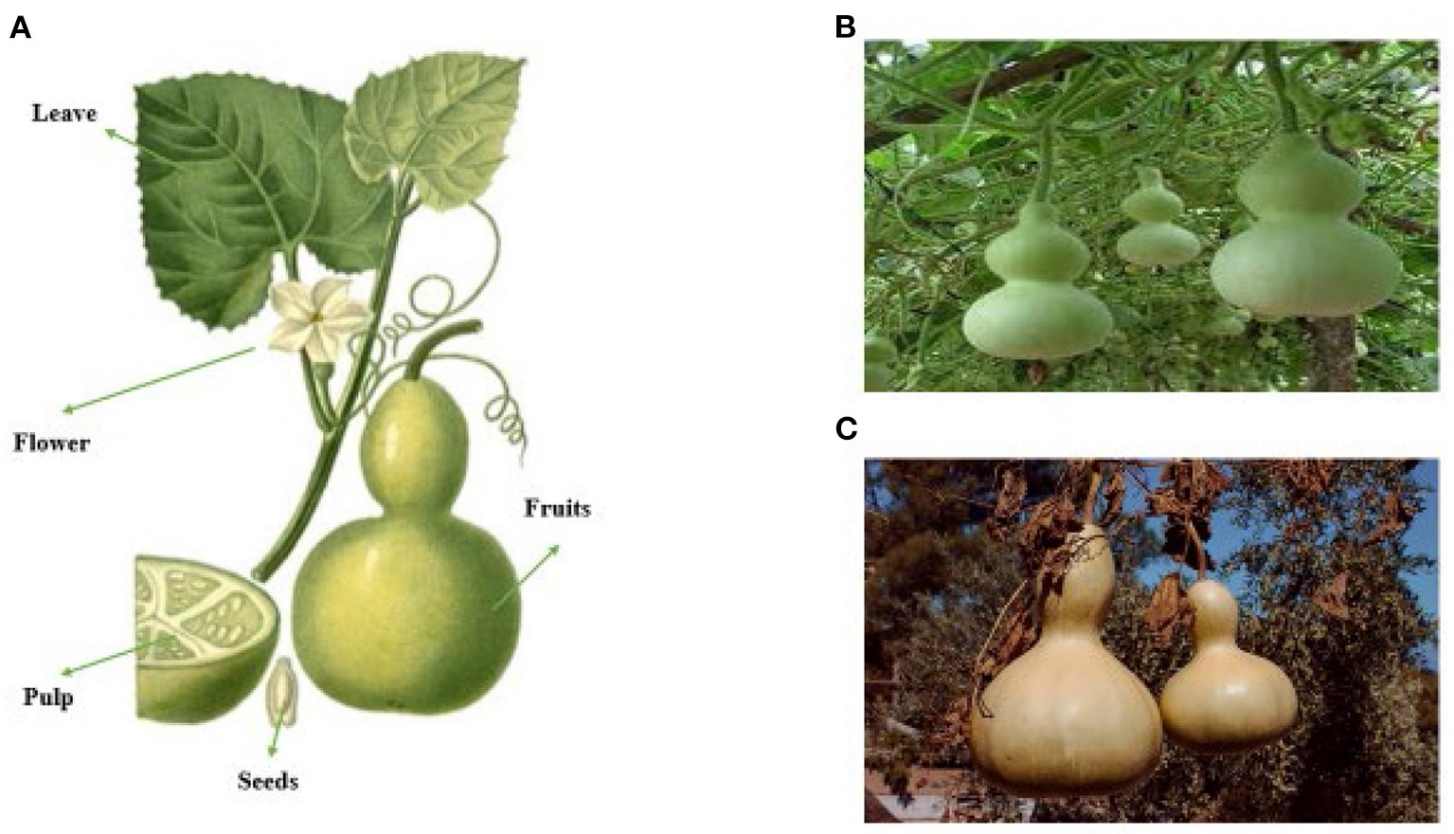
Various parts of *Lagenaria siceraria* **(A)**, fresh fruit of LS **(B)**, ripened fruit of LS **(C)**.

Figure 2 shows different parts of *Lagenaria siceraria* (A), fresh fruit (B), ripened fruit (C).

## Vernacular names

Vernacular names of *L. siceraria* in different languages across the world are given in Table 2.

**Table 2:** Vernacular names of *Lagenaria siceraria* (8).

**Language**	**Names**
Sanskrit	Katutumbi, Tumbi Ishavaaku, Tiktaalaabu, Alobu, Alaabu
Bengal	Loki, Laus, tumbi
English	Bottle gourd
Malayalam	Churan, Tumburini, Choraikka, Tumburu, Piccura, Chorakka, cura
Kannada	Isugumbala, Tumbi
Hindi	Lauki, Ghiya
Gujarati	Dudi, Tumbadi
Telugu	Sorakaya, Anapakaya
Urdu	Ghiya, Lauki, Kadu
Tamil	Sorakkai, Surai, Sorakkai
Marathi	Phopla
Punjabi	Tumbi, Dani

## Traditional uses

The fruit is extensively used as a medicinal vegetable in Asia and Africa for a variety of ailments. Alternative medicine is made from several components of this plant, including the fruit, seed, leaf, and root (9). In Ayurveda and other folk remedies, the plant's fruits had been noted to have possible therapeutic benefits. Traditional uses of the fruit include cardioprotective, antidote, aphrodisiac, cardiotonic, diuretic, and general tonic properties (5). The fruit juice had been a cure for jaundice and heal other liver ailments as it possessed good anti-oxidants (10). Various properties that are beneficial for human health have been attributed to this plant like antioxidant, hypolipidemic, diuretic, laxative, hepatoprotective, analgesic, antihypertensive, cardioprotective, central nervous system stimulant, anthelmintic, free radical scavenging, immunosuppressive, and adaptogenic (11). Anti-HIV, antipyretic, anthelmintic, anxiolytic, carminative, anti-diabetic, antibacterial, antioxidant, laxative, anti-tuberculosis, anti-diarrhoeal, and purgative are only a few of the therapeutic qualities of the Cucurbitaceae family. It's also used as a contraceptive, diuretic, and cardiotonic agent. Anti-inflammatory, antitussive, cytotoxic, and expectorant activities are also present (3). The diuretic efficacy of methanol and vacuum dried juice extract of the fruits had been studied. Albino rats had a larger urine volume when compared with the control group. Both of the extracts displayed a dose-dependent rise in electrolyte excretion (12). The plant species aids in improved digestion eliminates urinary difficulties, and aids in weight loss and blood pressure-lowering (13). For the treatment of illnesses and disorders in humans, *L. siceraria* has been utilized in several systems of traditional medicine. This vegetable has high water content and a low-calorie count. The seeds are also utilized for headaches and constipation since they have a cooling impact on the body (8).

After drying the fruit is used as resonance boxes for the kora and balafon (xylophone). Drinking water, milk, liquor, local wine, oatmeal, food grains, animal fat, honey, tobacco, ghee, salt, perfume, medicinal herbs, and crop seeds are all stored and transported in dried bottle gourd fruits. Beehives, beer-making containers, or storing clothing and cutlery are all created from dried fruit shells. Many musical instruments and beautiful decorations are made from dried bottle gourds (14). The climber's medicinal abilities have been used to cure a variety of disorders, including jaundice, ulcers, colitis, diabetes, insanity, skin problems hypertension, piles, and congestive cardiac failure (CCF). The fruit pulp has cooling, diuretic, antibilious, and pectoral qualities, and is used as an emetic and purgative. This pulp boiled in oil is used in treating rheumatism and sleeplessness (15).

Traditional uses of various parts of *L. siceraria* are given in Table 3 (16).

**Table 3:** Traditional uses of different parts of *Lagenaria siceraria* (16).

**Plant part**	**Traditional use**
Fruit pulp	Emetic, purgative, cooling, antibilious, sedative, diuretic
Flowers	Poison antidote
Stem bark and rind	Diuretic
Leaf juice	Hair growth, tooth decay, heart diseases, urinary disorders, jaundice, digestive disorders, constipation, diabetes, and cooling effect.
Seed	Vermifuge
Leaves	Purgative

## Phytochemistry

Ascorbic acid, triterpenes, minerals, choline, amino acids, vitamin-B complex, triterpenoid cucurbitacins B, D, H, G, 22-deoxy cucurbitacin, β-glycosidedase-elasterase, flavonoids, sterols, and carbohydrates are all found in the edible part of the fruit (5).

Cucurbitacins B, H, G, and D, as well as the bitter principle of the Cucurbitaceae, are said to be present in the fruit along with Flavone-C glycosides (a ribosome-inactivating protein), two sterols, i.e., fucosterol and campesterol, terpene byonolic acid (an allergic compound) and Lagenin (1).

**Figure 3:**
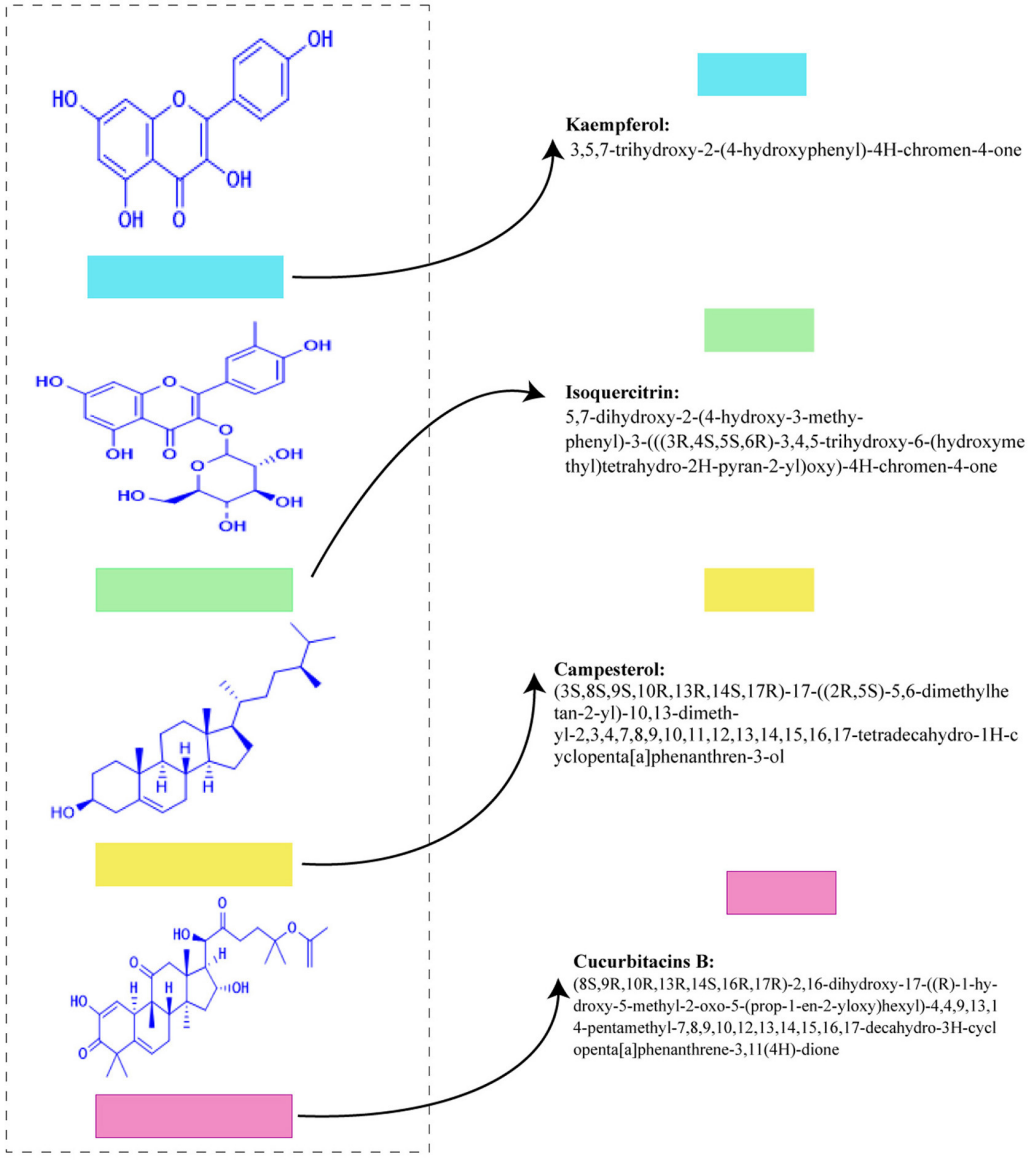
Some of the structures of different bioactive chemicals present in *Lagenaria siceraria*.

**Figure 4:**
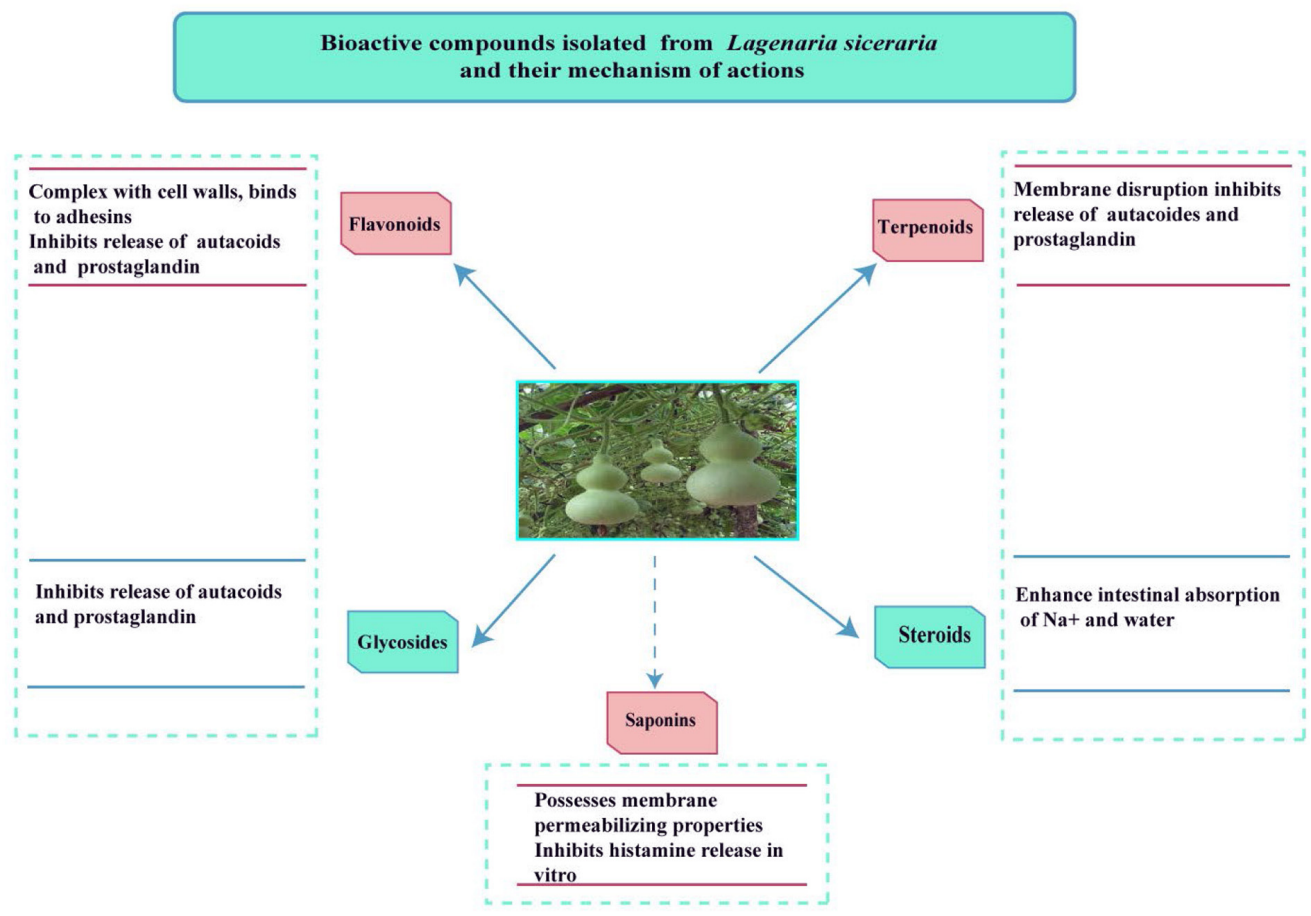
The action mechanism of various phytochemicals.

Figures 3, 4 shows the phytochemicals of *Lagenaria siceraria*.

The extract has carbohydrates, saponins, proteins, flavonoids, and glycosides as shown by the phytochemical test (17). This vegetable has high water content and a low-calorie value. Vitamins, choline, flavonoids, minerals, proteins, terpenoids, and other phytochemicals are found in the edible section. *L. siceraria* contains a variety of bioactive chemicals, including flavones, sterols, cucurbitacins, C-glycosides, triterpenoids, and -glycosides (2).

Table 4 shows phytochemicals and their functions present in different parts of *Lagenaria siceraria*.

**Table 4:** Phytochemicals and their functions present in different parts of *Lagenaria siceraria*.

**Phytochemical**	**Present in**	**Function**	**References**
Cucurbitacins H, D, B, G fucosterol and campesterol (Two sterols) Flavone-C glycosides	Fruit	Antimicrobial Antidiarrhoeal	(1, 18, 19)
Flavonoids, carbohydrates, proteins, glycosides, and saponins	Plant extract		(17)
Glucose and fructose and traces of sucrose	Whole fruit		(20)
Ascorbic acid, minerals, vitamin-B complex, β-carotene, choline, amino acids, 22-deoxy cucurbitacin, triterpenoid, cucurbitacins B, D, G, H, flavonoids, β-glycosidedase-elasterase, sterols, and carbohydrates, C-glycosides, triterpenes, and β-glycosides.	Fruit	Antimicrobial Antidiarrheal	(2, 5, 19)
Flavonoids, triterpenoid, sterols, β sitosterol, campesterol, isoquercitrin, and kaempferol	Methanolic extract	Antimicrobial Antidiarrhoeal Antihyperlipidemic	(19, 21, 22)
Triterpenoids (22- Deoxocucurbitacin D and 22- Deoxoisocucurbitacin D)	Fruit	Antimicrobial Antidiarrheal	(23, 24)

## Nutritional profile

Seeds contained 45.0–47.8 g/100 g crude fat, 8.1–7.3 g/100 g carbohydrates, 37.2–35.0 g/100 g crude protein, and 4.0 g/100 g moisture (25).

### Nutrients

The nutrient composition of *L. siceraria* (fruit and seeds) is given in Table 5.

**Table 5:** Proximate analysis of fruit and seeds of *Lagenaria siceraria* (11, 26).

**Nutrients**	**Fruit (in 100 g of edible portion)**	**Seeds (%)**
**Proximate composition**		
Carbohydrate	2.5 g	45.93
Protein	0.2 g	8.93
Fat	1.0 g	38.92
Fiber	0.6 g	
Energy	12 calorie	
Mineral	0.5 g	3.5
Moisture	96.1 g	2.72

The USDA (United States Department of Agriculture) nutritional database exhibited that each 100 g of *L. siceraria* has 14 Kcal energy, 3.39 g carbohydrates, 0.62 g protein, 0.2 g fat, and 0.5 g fiber (27).

## Minerals

Calcium, Potassium, Magnesium, Lead, Iron, Sodium, Zinc, and chromium were found in the seeds of *L. siceraria* fruit (26). Furthermore, Phosphorus, Copper, Manganese, and Cobalt were also reported in this plant (28).

Table 6 shows numerous minerals present in *L. siceraria*.

**Table 6:** Mineral composition of *Lagenaria siceraria* (USDA nutritional database).

**Mineral**	**Value in mg**
Sodium	2
Potassium	150
Magnesium	11
Copper	0.034
Phosphorus	13
Calcium	26
Manganese	0.089
Iron	0.20
Selenium	0.2
Zinc	0.70

## Amino acids

Seventeen amino acids lysine, methionine, threonine, proline, cysteine, glutamic acid, phenylalanine, arginine, tyrosine, histidine, valine, serine with glutamic acid, alanine, leucine, isoleucine, aspartic acid, glycine, leucine, and aspartic acid were found in seeds of *L. siceraria* (28).

## Health effects of *L. siceraria*

### Anti-inflammatory properties

In rats and mice, *L. siceraria'*s ethanolic extract (fruit and leaves) was tested for anti-inflammatory and analgesic properties. Carrageenan-induced edema, tail immersion pain, and acetic acid-induced writhing models were used to investigate the extract's activity. On the writhing test, the extract exhibited strong anti-inflammatory and analgesic potential. The extract comprises flavonoids, carbohydrates, proteins, glycosides, and saponins, according to a phytochemical analysis (17).

### Anti-oxidant properties

The aqueous extract of *L. siceraria* has a strong scavenging action, and the high phenolic content of calabash fruit may help to alleviate the oxidative stress associated with diabetes (29). Antioxidant activity is recognized for phenolics and flavonoids, which have a remarkable capacity to scavenge free radicals created in human bodies. As a result, determining the number of phenolics and flavonoids in a plant sample can help determine its antioxidant capacity. The antioxidant capacity of the pedicles of *L. siceraria* fruits was investigated *in vitro*. The ethyl acetate fraction even at low concentrations showed the most effective DPPH radical scavenger (30). *In vitro*, a methanol extract of the aerial section of *L. siceraria* was reported to scavenge DPPH, hydrogen peroxide, superoxide radical, and nitric oxide as well as prevent lipid peroxidation in a concentration-dependent way. The antioxidant action of MELS (methanol extract of *L. siceraria* aerial parts) was attributed to its high phenolic and flavonoid content (31). *In vitro* experiments such as the reducing power assay, radical scavenging assay, superoxide scavenging assay, lipid peroxidation inhibition assay, and the ethyl acetate extract of bottle gourd were shown to exhibit high antioxidant activity. The quantity of phenolic compounds found in bottle gourd extracts is proportional to their radical scavenging action (32). In an isolated rat heart model, the extract's antioxidant capacity was measured in terms of glutathione peroxidase (GPx), catalase (CAT), superoxide dismutase (SOD), vitamin C (Vit C), glutathione reductase (GR), reduced glutathione (GSH), vitamin E (Vit E), and glutathione S-transferase (GST). The activities of enzyme antioxidants such as CAT, GSH, GR, and SOD were significantly reduced in isoproterenol-induced rats. It may be determined that *L. siceraria*'s ethanolic extract has antioxidant effects (33). Extraction of seeds with ethanol resulted in a significant number of phytochemicals and antioxidant activity. All of these phytochemicals are powerful reducing agents, metal chelators, and radical scavengers, and they may be to blame for the seeds' high antioxidant activity. The methanolic extract of seeds showed good DPPH and radical scavenging capabilities in antioxidant tests (34). In human patients with dyslipidemia, the effects of *L. siceraria* fruit extract were investigated. The antioxidant potential of *L. siceraria* fruit extract was demonstrated in dyslipidemic patients by increases in Superoxide dismutase and Glutathione levels (35).

### Anti-cancer properties

**Figure 5:**
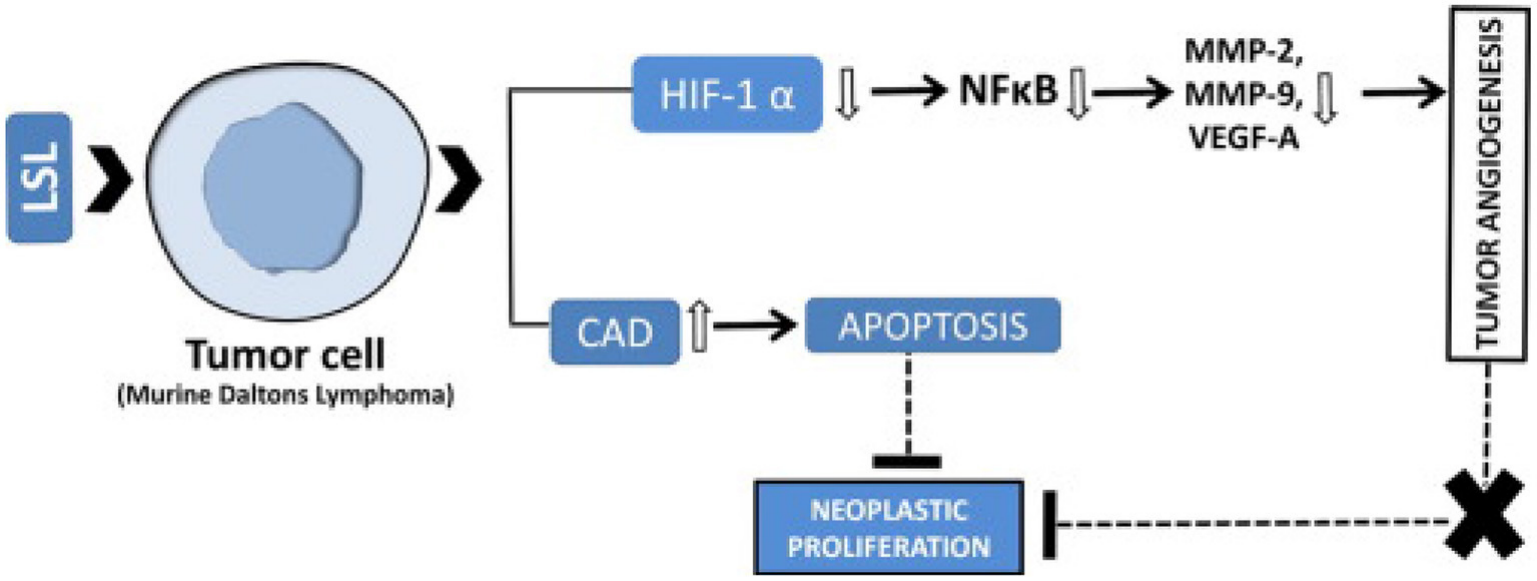
Anti-cancerous mechanism of action of *Lagenaria siceraria* (41).

The study aimed to see the effect of methanol extract of *L. siceraria* aerial parts on anti-cancer properties. In mice, Ehrlich's Ascites Carcinoma model. The effect of medication response was assessed using the research of tumor growth response, which included an increase in life duration, a study of hematological parameters, biochemical estimates, and a liver tissue antioxidant assay. The cytotoxicity and antioxidant capabilities of *L. siceraria*, as well as the flavonoid content of the methanol extract of *L. siceraria* aerial parts, demonstrated that *L. siceraria* has strong anticancer activity (36). The antitumor effectiveness of *L. siceraria* fruit was investigated using human cancer cell lines (MCF-7 and HT-29). With varied potency and selectivity, the bitter component of *L. siceraria* displayed substantial anticancer action against both cancer cell lines. Cucurbitacin I and other bioactive compounds in *L. siceraria* fruit bitter extracts had dose-dependent inhibitory and cytotoxic effects on tested cell lines, which can be ascribed to the presence of cucurbitacin I and other bioactive compounds in *L. siceraria* fruit bitter extracts (34). A methanolic extract of *L. siceraria* Standley Fruit was tested for anti-mutagenic properties. The anti-mutagenicity of plant extracts ranged from low to high. The Ames test was employed in this investigation to assess the antimutagenic activity of direct (Sodium azide) acting mutagens in *Salmonella typhimurium* strains TA98 and TA100. The study found that the TA98 and TA100 strains have considerable antimutagenicity against mutagen. The antimutagenicity of the extract discovered in this investigation suggests that *L. siceraria* Standley Fruit has chemopreventive pharmacological relevance, which is attributable to its anti-oxidant potential (37). In Swiss albino mice, the chemopreventive efficacy of bottle gourd juice (BGJ) against cutaneous papilloma genesis was investigated. The chemopreventive properties of bottle gourd against skin cancer were demonstrated by a reduction in papilloma number, multiplicity, incidence, latency, volume, and papilloma size, as well as histological examinations. The presence of phytochemicals working through several pathways might be responsible for the protective benefits (38, 39). On lung cancer cell lines, the cytotoxic effects of plant fruit extract were investigated. According to the findings, the presence of cucurbitacin, polysaccharide inhibitor lagenin, and flavonoids in this plant's extract had a significant growth inhibitory influence on the lung cancer cell line (40). Immune-potentiating action is induced by the latex sap of dietary *L. siceraria* (LSL), which has high lectin activity. In both *in vitro* and *in vivo* tumor models, LSL inhibits tumor cell growth. Through changed gene expression, it suppresses tumor growth by targeting apoptosis and tumoral neovasculature. Its possible mechanism of action is given in Figure 5 (41).

### Anti-obesity properties

Some fatty acid esters were found in the chloroform fraction of *L. siceraria*, including isopropyl palmitate, 9,12- octadecadienoic acid methyl ester, hexadecanoic acid methyl ester, and alpha-linolenic acid methyl ester. *L. siceraria's* ability to decrease pancreatic lipase activity, reducing lipid breakdown and hence lowering fat entrance into the body, is due to these chemicals. Regular consumption of the fruit's aqueous decoction may therefore be suggested for weight loss. Fatty acids and their esters acted as lipase inhibitors (42). In high-fat diet-generated obese mice, the synergistic impact of *Commiphora Mukul* (Gum Resin) with *L. siceraria* (fruit) extracts was examined. After the combined treatment, there was a substantial reduction in body weight, triglyceride, VLDL levels, fasting blood glucose, LDL, and serum cholesterol, as well as an increase in HDL levels. The results showed that combining *C. Mukul* and *L. siceraria* reduced obesity caused by a high-fat diet (43). The effects of *L. siceraria* fruit extract on human disease patients were investigated. There were significant decreases in body mass index (35). Obesity in Wistar albino rats was created by feeding them a high-fat diet and were treated with a diet containing *L. siceraria*, there was a significant reduction in body weight, locomotor activity, total cholesterol, food intake, triglycerides, organ weights, and an increase in low and high-density lipoprotein levels, indicating that *L. siceraria* has anti-obesity potential. The aqueous extract included numerous chemical ingredients such as saponins, pectin, and ellagic acid, which are essential for decreasing body weight and cholesterol levels, according to the preliminary phytochemical assessment of LS and TA. The LS fruits are high in saponins, cucurbitacins B, G, D, H, triterpenoid, and pectin, which showed lipid-lowering properties (44, 45). Different bottle gourd extracts were tested for their antihyperlipidemic and hypolipidemic properties in Triton-induced hyperlipidemic rats, as well as their hypolipidemic effects in normocholesterolemic rats. The extracts lowered total cholesterol, triglycerides, and low-density lipoproteins levels in a dose-dependent manner, while dramatically increasing high-density lipoproteins levels. The effects of petroleum ether extract were not significant. When compared to the others, the chloroform and alcoholic extracts had more substantial impacts on triglycerides, total cholesterol, and low-density lipoproteins, as well as an increase in HDL (46). The antihyperlipidemic activity of a methanolic extract of *L. siceraria* fruits (LSFE) was tested in high-fat-diet-induced hyperlipidemic rats. When compared to rats fed a high-fat diet, the gain in weight in rats given LSFE was smaller. Furthermore, LSFE caused a considerable increase in bile acid excretion. It may work by affecting endogenous cholesterol production in the liver and boosting cholesterol end product excretion. The LSFE included flavonoids, saponins and steroids, and polyphenolics, according to preliminary phytochemical screening. Plant saponins and steroids have been shown in several investigations to have hypolipidemic and antihyperlipidemic properties (47). The juice of *L. siceraria* (Bottle gourd) includes all of the active components that inhibit fat storage in adipose tissue. The anti-obesity activity of *L. siceraria* (Bottle gourd) juice has been tested in overweight and obese human individuals. Weight, waist circumference, and BMI all decreased significantly in those who consumed Bottle gourd juice, indicating it is a safe and effective treatment option for obese people (48).

### Immunity boosting properties

The immunomodulatory impact of a methanolic extract of *L. siceraria* fruits in rats was investigated. The different fractions of *L. siceraria* were given orally at dosages of 100, 200, and 500 mg/kg to rats resulting in a great reduction in the delayed-type hypersensitivity reaction. Both primary and secondary antibody titers increased in a dose-dependent manner. Fractions also enhanced the number of white blood cells and lymphocytes. The findings show that test fractions have immunomodulatory potential (49).

### Anti-diabetes properties

*In vitro*, the aqueous fraction of *L. siceraria* fruit pedicles has significant alpha-amylase inhibitory activity. This exercise is employed as a blood glucose management method. The conversion of starch to glucose is slowed when pancreatic alpha-amylase is blocked in the small intestine. As a result, less glucose is generated and enters the bloodstream, allowing it to be employed as an anti-diabetic drug (30). Supplementing MELS enhanced lipid metabolism and functions as a preventive mechanism against the formation of atherosclerosis in diabetic rats, as well as reducing diabetic sequelae from lipid peroxidation by boosting antioxidant status. As a consequence, the aerial sections of *L. siceraria* methanol extract can be considered a rich source of anti-diabetic medicines, possibly due to the extract's flavonoid and polyphenolic content (50). The pulp and seed extracts of *L. siceraria* induced alterations in the functional status of pancreatic cells. In rats with alloxan-induced diabetes, the capacity of the organism to generate and release insulin rose at the same time as the glucose level in the blood declined. The pulp and seed extract of *L. siceraria* were shown to have considerable anti-diabetic action (51). The oral glucose tolerance test was used to assess the hypoglycemic qualities of different globulins extracted from male Wistar rats, and it revealed that *L. siceraria* seeds contained globulins with high anti-hyperglycemic activity. In the profile, there was a prominent protein band with a molecular weight of 24.61 kDa that had considerable anti-hyperglycemic action. This specific protein, if present, is most likely the active peptide responsible for the observed activity (52). In human patients with dyslipidemia, the effects of *L. siceraria* fruit extract were investigated. Fasting blood glucose levels were found to be significantly lower (35). Various *in-vitro* approaches, such as amylolysis kinetics, and glucose adsorption diffusion capacity, were used to assess the hypoglycemic efficacy of the phyto-material extracts. The suppression of an enzyme (alpha-amylase) by *L. siceraria*, which restricts starch to glucose conversion, was blamed for the slowing of glucose diffusion. The extracts of *L. siceraria* have been shown to have hypoglycemic action in several *in-vitro* tests and might be employed as therapeutic agents in the treatment of diabetes (53). The presence of bioactive molecules and amylase and glucosidase inhibitors, and cholinergic esterase enzymes in ethanol and methanol seed extracts of *L. siceraria* might explain the excellent antidiabetic action found (34).

### Cardio-protective properties

Ethanolic extract has a cardioprotective effect. The antioxidant function of *L. siceraria* (Mol) fruits is most likely due to its capacity to combat free radicals, or its ability to maintain the near-normal activity of free radical enzymes, which protect the cardiac membrane from oxidative damage by lowering lipid peroxidation (33).

Modern pharmacological research has revealed that the fruit of *L. siceraria* has a variety of cardioprotective characteristics. In rats with triton-induced hyperlipidemia, chloroform, and alcoholic extracts of *L. siceraria* revealed antihyperlipidemic potential. In Doxorubicin and Isoproterenol-induced cardiotoxicity in rats, the fruit demonstrated strong cardioprotective benefits (5). The goal of the study was to see if *L. siceraria* (LS) fruit powder might protect rats against the cardiotoxicity of the drug doxorubicin (Dox). The LS-treated group was shown to be protected from doxorubicin-induced cardiac damage in histopathological analysis. It was discovered that *L. siceraria* had a cardioprotective effect in rats when they were exposed to doxorubicin-induced cardiotoxicity (54). The lack of cardiac necrosis and inflammation in the *L. siceraria*-treated group showed that the plant had cardioprotective properties. The antioxidants orientin and isoorentin found in *L. siceraria* fruit powder appear to help prevent cardiac necrosis and inflammation. As a result, it may be inferred that LS fruit has cardioprotective properties (55). By preserving endogenous antioxidants and reducing lipid peroxidation in the rat heart, *L. siceraria* (Cucurbitaceous) Fruit Juice reduced Doxorubicin-induced cardiotoxicity and lowered myocardial damage (56). In isoproterenol-induced myocardial infarction, the cardioprotective benefits of *L. siceraria* fruit juice were investigated. The results show that *L. siceraria* fruit juice has a cardioprotective effect in rats with isoproterenol-induced myocardial infarction. The presence of polyphenolic components in LS fruit may be responsible for these effects (57). Isoprenaline-induced tachycardia was decreased when *L. siceraria* fruit powder was given. Isoprenaline cardiotoxic impact was reduced in LS-pretreated mice. The cardioprotective effect of LS in isoprenaline-induced cardiotoxicity appears to be aided by its antioxidant and anti-inflammatory properties (58).

### Gastro-protective properties

The anti-ulcer efficacy of a methanolic extract of *L. siceraria* fruits was examined in Wistar rats using pylorus ligation, Asprin, cold-restraint stress, and ethanol ulcer models. MELS reduced stomach volume, free acidity, ulcer index, and total acidity significantly, indicating that *L. siceraria* fruit extract may have anti-ulcer action (59). *L. siceraria* showed an increase in Gastric juice pH, whereas decreased in Total acidity, Gastric content, and Gastric juice volume. As a result of histological assessment investigations, it was shown that *L. siceraria* is both safe and effective in the treatment of stomach ulcers (60).

### Hepato-protective properties

Based on improvements in serum marker enzyme levels, antioxidant parameters, and histological investigations, ethanolic extract of *L. siceraria* fruit is said to have a high hepatoprotective and antioxidant effect in antitubercular drugs induced hepatotoxicity (61).

*L. siceraria* has been shown to prevent the elevation of hepatic enzymes caused by long-term carbamazepine administration in rabbits, as well as liver tissue histology showing no necrosis or cholestasis. Thus, it is concluded that *L. siceraria* has a hepatoprotective effect and reduces the hepatotoxicity caused by carbamazepine (62). On rats, the hepatoprotective efficacy of *L. siceraria* fruit extracts was evaluated against carbon tetrachloride (CCl_4_)-induced hepatotoxicity. The toxic effect of CCl_4_ was dramatically reduced in *L. siceraria* ethanol extract-treated rats by restoring serum bilirubin, protein, and enzyme levels. The existence of normal hepatic cords, lack of necrosis, and fatty infiltration in the liver sections of the animals treated with the extracts further demonstrated the hepatoprotective potential (63). In paracetamol-induced hepatotoxicity in rats, the presence of phenolic components in ethanol extract of *L. siceraria* fruit protected against oxidative damage and liver necrosis (10).

### Other pharmacological effects

*L. siceraria* (Molina) Standl. is a traditional medicinal as well as a portion of vegetable food. Immunomodulatory, antioxidant, hepatoprotective, anti-stress, cardioprotective, adaptogenic, anti-inflammatory, antihyperlipidemic, and analgesic activities have all been described. Lagenin (20 kDa), a new protein isolated from seeds, has been shown to have anticancer, antiviral, antiproliferative, and anti-HIV properties (64). Antioxidant, antihypertensive, hepatoprotective, cardioprotective, laxative, diuretic, central nervous system stimulant, adaptogenic, immunosuppressive, hypolipidemic, analgesic, anthelmintic, and free radical scavenging properties have also been proposed for the plant. This pulp boiled in oil is used in treating rheumatism and sleeplessness (16). Polysaccharides extracted from various portions of the plant have been found to have immune-modulating, anti-inflammatory, anti-tumor, cardioprotective, antioxidant, hepatoprotective, anti-diabetic, anti-hyperlipidemic, and analgesic activities in the last three decades. Several polysaccharides isolated from diverse sections of *L. siceraria* have been proposed with a variety of structures. Apart from its various bioactive qualities, this plant has the capacity to detoxify soil from heavy metals through the process of bioremediation (4). Antianxiety, antihyperlipidemic, diuretic, cytotoxic, cardioprotective, anti-inflammatory, antiulcer, analgesic, anticancer, antimicrobial, antidepressant, anti-hyperglycemic, anthelmintic, anti-urolithiatic, hepatoprotective, anthelmintic, immunomodulatory, antistress, and antioxidant activities have been studied in various parts of this plant (fruit, leaves, flowers, and roots) (2). Antimicrobial activity of *L. siceraria* extracts against *Enterococcus faecalis, Salmonella typhi, Staphylococcus aureus, Klebsiella pneumonia, E. coli*, and antifungal strains (*Aspergillus flavus, Trichoderma harzianum*, and *Aspergillus oryzae*) was moderate to strong (65). Fruits of *L. siceraria* have the ability to promote bile salt excretion, and their supplementation lowered fat levels in rats over time (47). Avinash et al. (66) described the antiulcer effect of *L. siceraria*. Long-term administration of *L. siceraria* fruit powder was done in dexamethasone-induced rats. According to the findings, hypertension activity in rats has decreased (58). *L. siceraria* crude methanol extract has antihelmintic efficacy against the Indian earthworm *Pheretima posthuma*. As a result, the leaves of *L. siceraria* are thought to have significant anthelmintic action (32). Adedapo et al. (67) explored the effects of the leaf extract on carrageenan- and histamine-induced paw edema in rats with swollen paws. In addition, the authors used mice to conduct acetic acid writhing and formalin tests. In rats, the scientists found a substantial reduction in paw edema, licking duration, and frequency. It has antihyperlipidemic, cardioprotective, hepatoprotective, diuretic, antidiabetic, and antihyperlipidemic effects (8). Antioxidant, antimicrobial, central nervous system activity, bronchospasm protective, antihyperglycemic, antidiarrheal activity, hepatoprotective activity, analgesic, antihelmintic activity, anti-inflammatory activity, cardioprotective effects, cytotoxic, antidiabetic, anticancer, antihyperlipidemic activity, immunomodulatory effect, and diuretic activity were discovered in the plant (68). The analgesic efficacy of methanolic and aqueous extracts of the fruit was investigated using the tail immersion method in rats to provide scientific validity to the folkloric medicinal usage of *L. siceraria*. The methanolic extract has a moderate activity, whereas the aqueous extract has a high activity, according to the pain threshold test. This backs up the plant's historic usage in painful and inflammatory illnesses (69). Zinc oxide nanoparticles made from *L. siceraria* extract were reported for antimicrobial, anti-dandruff, and anti-arthritic properties (70).

**Figure 6:**
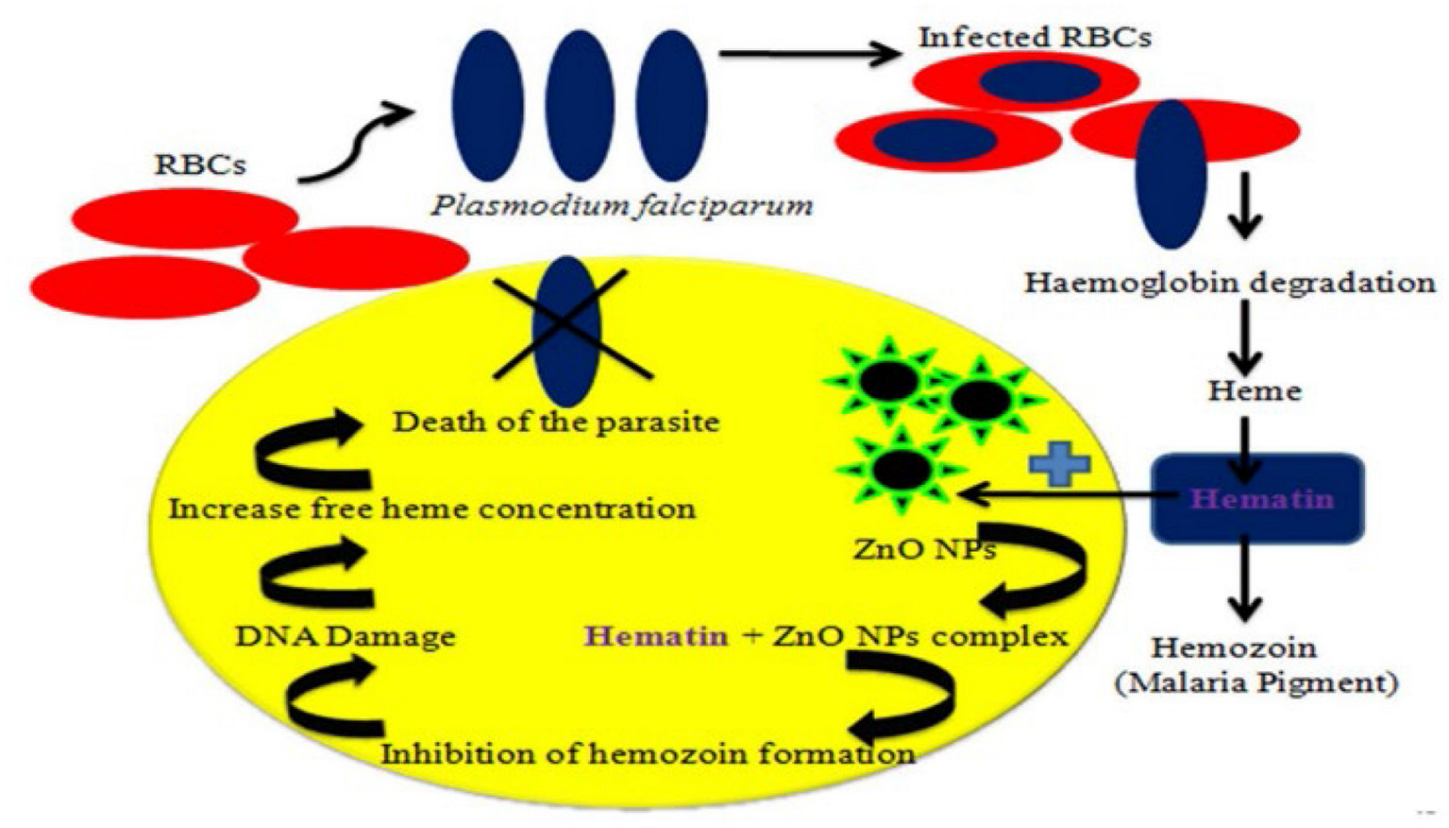
Schematic representation of the mechanism of ZnO NPs (nano-particles from LS) on *Plasmodium falciparum* (73).

Various extracts from the leaves and stems of *L. siceraria* were tested for their ability to repel the *Culex pipiens* L. mosquito, and it was concluded that these extracts could be developed as commercial products as an effective protection measure against mosquito bites, and thus control infections transmitted by a mosquito (71). The activity of *L. siceraria* leaves aqueous extract and silver nanoparticles (AgNPs) produced by *L. siceraria* against immature stages of *C. pipiens* and *A. phronesis* was examined. For 24 h, immature stages of both mosquito species were exposed to 400, 300, 200, 100, and 50 ppm aqueous extract of *L. siceraria* leaves and 5, 10, 20, 30, and 40 ppm *L. siceraria* generated AgNPs. The results exhibited that, AgNPs generated by *L. siceraria* were more harmful to mosquito species examined than the aqueous extract of *L. siceraria* leaves (72). The effect of manufactured zinc oxide nanoparticles (ZnO NPs) made from zinc nitrate and aqueous peel extract of *L. siceraria* on malaria prevention was investigated. The extract of *L. siceraria* and its mediated ZnO NPs were tested on *An. stephensi* III instar larvae. The influence of the ZnO NPs-based therapy on the histology and morphology of mosquito larvae was also studied. *Poecilia reticulata* (*P. reticulata*) had a 44% predation efficiency against *An. Stephensi* larvae in a normal laboratory environment, but 45.8 and 61.13% predation efficiency against *An. Stephensi* larvae in aqueous *L. siceraria* extract and its mediated ZnO NPs contaminated environment, respectively. ZnO NPs produced by *L. siceraria* were tested against *Plasmodium falciparum* CQ-sensitive strains. With an IC50 value of 62.5 g/mL, the *L. siceraria* extract and its induced ZnO NPs displayed cytotoxic effects against HeLa cell lines. According to the findings, *L. siceraria* peel extract and *L. siceraria*-produced ZnO NPs are viable green choices for fighting malarial vectors and parasites (73). Figure 6 shows the mechanism of ZnO NPs on *Plasmodium falciparum*.

Using a forced swim (behavior despair) paradigm, the antidepressant effect of methanolic extract of *L. siceraria* fruits in rats was assessed. The extract was given orally at dosages of 50, 100, and 200 mg/kg. The extract has antidepressant properties that are dosage dependent. The occurrence of triterpenoids, flavonoids, sterols, and saponins may be responsible for the action (23).

*L. siceraria* (LS) fruit juice has been used to treat jaundice and certain liver problems (10). The fruit is used to treat pain, ulcers, and fevers, as well as chest cough, asthma, and other bronchial problems, particularly in the form of a syrup made from sensitive fruits (1). Antioxidant, cardioprotective, hypolipidemic, diuretic, antihypertensive, hepatoprotective, analgesic, anthelmintic, free radical scavenging, immunosuppressive, central nervous system stimulant, laxative, and adaptogenic properties have also been proposed for the plant (11). Anti-inflammatory, antitussive, cytotoxic, and expectorant activities are also present in the plant (3). The plant species aids in improved digestion eliminates urinary difficulties, and aids in weight loss and blood pressure lowering (13). The seeds are used to treat headaches and constipation because they have a cooling impact on the body (8). The plant has long been renowned for its medicinal virtues, and it has been used to cure a variety of disorders, including diabetes, hypertension, ulcers, insanity, piles, jaundice, colitis, and skin infections. Its fruit pulp has cooling, diuretic, antibilious, and pectoral qualities, and is used as an emetic and purgative. This pulp is boiled in oil and used to treat rheumatism and sleeplessness (15).

**Figure 7:**
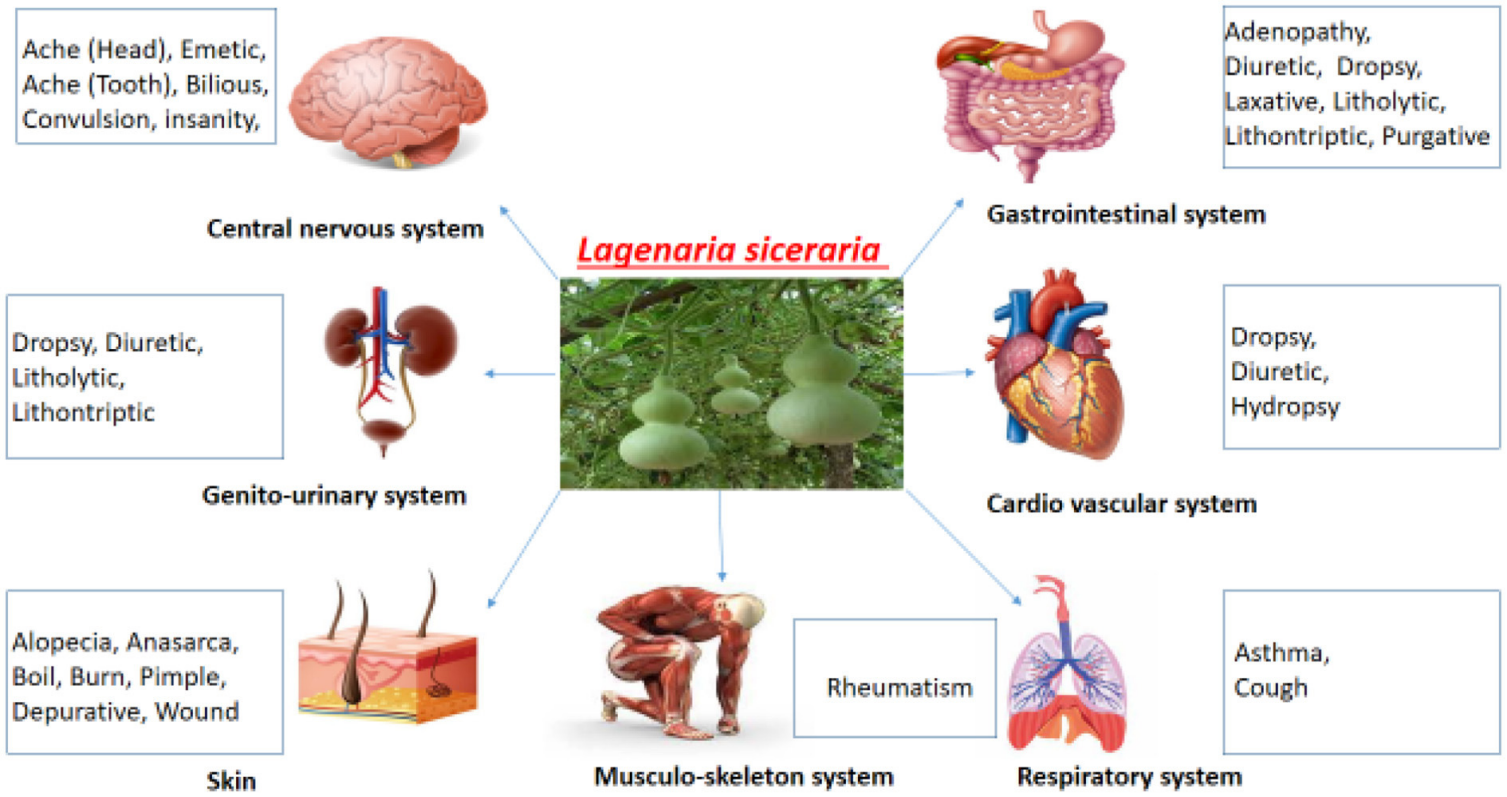
Systemic effects of *Lagenaria siceraria*.

The effect of *L. siceraria* on multiple systems of the human body is shown in Figure 7.

## Uses in poultry and veterinary

Medicinal plants are very popular to improve the health and productivity of farm animals (67–69). The *in vitro* anthelmintic activity of crude aqueous methanolic extract (CAME) of *L. siceraria* against *Haemonchus* (H.) *contortus* and their eggs was evaluated using an adult motility assay and an egg hatch test. The *in vivo* anthelmintic activity of various dosages of crude powder (CP) and CAME in sheep naturally infected with gastrointestinal helminths was investigated using the fecal egg count reduction assay. CAME has significant antihelmintic action *in vitro* and inhibited the hatching of *H. contortus* eggs (71). In sheep infected with Moniezia and Avitellina species, the anticestodal action of *L. siceraria* seeds was examined. The powdered seeds, as well as their extracts in water and methanol, were administered orally at various doses. On the 15th day following administration, the medication powder induced a decrease in egg per gram counts (23). The purpose of the study was to develop low-salt, high-fiber, and low-fat functional chicken nuggets by substituting bottle gourd for sodium chloride and observing the effects on physicochemical parameters textural, color values, lipid, and sensory properties of pre-standardized low-fat chicken nuggets. The results of this investigation revealed that substituting bottle gourd for sodium chloride has a substantial impact on a variety of product quality parameters. Salt substitution, on the other hand, did not affect sensory qualities. Excluding the taste and quality ratings, which were impacted at greater levels, the sensory qualities of low-salt, low-fat chicken nuggets with bottle gourd were equivalent to the Control. With the addition of bottle gourd, the dietary fiber content of the goods may be increased, while the total cholesterol level can be reduced. As a result, using this technique, extremely palatable low-fat, low-salt, and high-fiber functional chicken nuggets may be developed without sacrificing their acceptability (74).

## Toxicity assessment

*L. siceraria* is found to cause problems in the upper gastrointestinal system. The consumption of *L. siceraria* causes nausea, vomiting, gastrointestinal bleeding, abdominal pain, and hematemesis (20). It is also said to cause gastrointestinal toxicity along with gastric erosion (first stage of ulcer) and ulcers (21, 22). This toxicity may be attributed to the presence of triterpenoid cucurbitacins (24). This data is based on clinical data but no dose of toxicity has been mentioned. Recently, it has come to light that drinking bottle gourd juice with a bitter flavor, can have extremely hazardous responses and result in symptoms like abdominal discomfort, vomiting, diarrhea, shock, and death (75).

A little number of cucurbitacins, specifically the types including B, D, G, and H, are present in bottle gourd fruit. Cucurbitacin concentrations often don't go above 130 ppm (76). The binding of cortisol to glucosteroid receptor is inhibited by Cucurbitacins in He La cells at 37°C which depicts a strong correlation with cytotoxic activity (77). The capillary permeability is enhanced by Cucurbitacin D (78) which is associated with a persistent fall in blood pressure and accumulation of fluid in thoracic and abdominal cavities in mice.

## Conclusion and future perspective

The present review gives a thorough insight into *L. siceraria* phytochemistry along with pharmacology, beneficial effects, medicinal uses, and limitations that suggest its' therapeutic potential. The *L. siceraria* has various critical health-promoting benefits such as neurological, physiological, and blood biochemical changes. Though the mechanism of action for phytochemicals may differ among various species and is not fully understood, therefore, need to be exploited. Further research is also warranted to uncover and record relevant markers (bio and molecular) that are responsible for a wide range of *L. siceraria* health benefits in humans, animals, and poultry.

## Author contributions

MS and MK drafted the article. JB, KA, AM, and MA downloaded the articles. UY, AK, and ZM edited the article, while SC gave the main idea. All authors contributed to the article and approved the submitted version.

## Conflict of interest

The authors declare that the research was conducted in the absence of any commercial or financial relationships that could be construed as a potential conflict of interest.

## Publisher's note

All claims expressed in this article are solely those of the authors and do not necessarily represent those of their affiliated organizations, or those of the publisher, the editors and the reviewers. Any product that may be evaluated in this article, or claim that may be made by its manufacturer, is not guaranteed or endorsed by the publisher.
